# Validation of Sleep Bruxism Questionnaire Toward the Experience of Jaw Pain and Limitation of Jaw Movement in Saudi Arabian Adolescents

**DOI:** 10.7759/cureus.26120

**Published:** 2022-06-20

**Authors:** Hammam A Bahammam

**Affiliations:** 1 Pediatric Dentistry, King Abdulaziz University, Jeddah, SAU

**Keywords:** sleep bruxism, pain, movement, joint, jaw, adolescent

## Abstract

Background: Bruxism in children and adolescents is underreported and needs strong awareness among parents. No study has attempted to validate the sleep bruxism questionnaire, particularly in Saudi adolescents.

Objective: This study aims to validate the sleep bruxism questionnaire on the experience of jaw pain and limitation of jaw movement in Saudi adolescents from 10 to 19 years of age.

Methods: This study was a cross-sectional (survey) study in which we distributed an Arabic translation of a questionnaire on sleep bruxism among 200 parents of patients who attended the dental clinics of the Faculty of Dentistry, King Abdulaziz University Hospital, Jeddah, Saudi Arabia, for treatment. Cronbach's alpha and interclass correlation coefficients were checked for reliability and internal consistency of the items. For validity, convergent validity analysis was accomplished by analyzing temporomandibular disorder and sleep bruxism. The response rate was 85%.

Results: There was a weak association between jaw pain with jaw lock (coefficient value = 0.249) and bruxism (coefficient value = 0.287). Bruxism and jaw lock had a robust correlation (coefficient value = 0.920).

Conclusion: The Arabic version of the sleep bruxism questionnaire is reliable and valid for assessing sleep bruxism toward the experience of jaw pain and limitation of jaw movement in Saudi Arabian adolescent patients.

## Introduction

According to international consensus, bruxism is the grinding or clenching of teeth or thrusting or bracing of the mandible due to repeated contraction activity of temporalis, masseter, or other jaw muscles [1]. It is categorized as primary or secondary depending on the absence of association with other clinical disorders [2]. Bruxism can cause headache, hypertrophy of masticatory muscles, loss of tooth surface, tooth fractures, periodontal support loss, and hypersensitivity of teeth [3]. When bruxism occurs during sleep, it is called sleep bruxism [3]. Previously, sleep bruxism was considered a pathological or dysfunctional movement, which, after the revision of literature, is now considered a centrally controlled condition with various mediating risk factors [3]. It is caused by multiple pathophysiological (central), morphological (peripheral), and psychosocial (stress, anxiety, depression) risk factors in adults [4]. In children, disturbances in sleeping patterns and exposure to second-hand smoke were significant risk factors [5]. Sleep bruxism is a protective phenomenon in stimulating saliva flow during sleep or maintaining a clear airway. Schames et al. [6] observed that rhythmic masticatory muscle activity during sleep bruxism is an autonomic response of the body to slow down the heart rate to combat tachycardia caused by brain microarousals.

According to a review by Manfredini et al. [7], data on the prevalence of sleep bruxism are minimal and widely generalized. However, it is more prevalent in females [8]. Bruxism can be diagnosed through patient history (clinical interview) and examining and recording muscle activity [3]. A recent technology using artificial intelligence automatically identifies, quantifies, and characterizes sleep bruxism by analyzing mandibular movements [9]. Sleep bruxism is considered a normal physiological movement, and management of bruxism is required only if it causes secondary issues. Oral appliances are recommended as they may decrease muscle activity and prevent damage to dentition due to grinding or clenching.

Moreover, stretching of masticatory muscles has been linked to increased episodes and is ineffective in managing bruxism [10]. Irreversible occlusal adjustments in earlier studies showed no evidence of efficient management [3]. Improvement of sleep hygiene, biofeedback, and relaxation are some of the effective behavioral interventions. Moreover, applying botox (botulinum toxin) to the masticatory muscles can decrease the frequency of sleep bruxism. However, it is associated with severe side effects [11]. No psychological, pharmacological, or dental strategy has been entirely effective in curing sleep bruxism [11]. Bruxism is underreported in children and adolescents and needs strong awareness among the parents. However, according to Huynh et al. [12], parents who report clenching and grinding in their children are 83% accurate after the diagnosis is established. They further showed that sleep bruxism is highest during childhood, and the prevalence decreases with age. Therefore, it is more important to diagnose sleep bruxism in children and adolescents than children. Sleep bruxism causes secondary conditions, such as orofacial pain, headache, and pain on awakening [13]. Furthermore, no study has attempted to validate the sleep bruxism questionnaire, particularly in Saudi adolescents. This study aims to validate the sleep bruxism questionnaire based on the experience of jaw pain and limitation of jaw movement in Saudi adolescents from 10 to 19 years of age.

## Materials and methods

Research design and study sample

This study was a cross-sectional (survey) study. We distributed a questionnaire on sleep bruxism in Arabic translation among parents of patients who attended the dental clinics of the Faculty of Dentistry, King Abdulaziz University Hospital, Jeddah, Saudi Arabia, for treatment. The sample was obtained through convenience sampling and included adolescents from 10 to 19 years. Before participation, we provided an informed consent sheet and an information sheet explaining the aims and objectives of the study to the parents of the children. They also completed the questionnaire. We clarified that the participants could leave the study anytime. The study followed the Declaration of Helsinki for ethical considerations. The parents were asked to complete the questionnaire before the appointment. Factor analysis using a 10:1 patient-to-item ratio calculated sample size. As the model comprised 17 items, the study required a sample size of a minimum of 170. The study included male and female patients aged 10-19 years with a target sample size of 200. Ethical approval was obtained from the ethical committee of the King Abdulaziz University, Jeddah, Saudi Arabia, where the study took place.

Research questionnaire

We edited the questionnaire previously used to evaluate and report sleep bruxism to fulfill the objectives of this study [14-16]. The questionnaire consisted of three sections. Section 1 was demographics, which collected data on the age and gender of the adolescents and the employability status and educational status of parents. Section 2 questions were based on the criteria to diagnose sleep bruxism [17]. The questions (as demonstrated in the Arabic questionnaire) (Appendix 1) were based on the history of the past six months. Affirmative answers to questions 1 and 2 and one option from question 3 suggested sleep bruxism, whereas affirmative answers to question 3 were considered positive for jaw lock. Question 4 assessed jaw pain and severity, whereas question 4 was a visual analog scale (VAS) to report the severity of pain. Part 3 had eight questions, which had a four-point rating (never = 0, sometimes = 1, often = 2, always = 3).

For temporomandibular disorder (TMD) diagnosis, Index Diagnostic Temporomandibular Disorder (ID-TMD) questionnaire was used for translation [18]. We evaluated the Arabic translation of the questionnaire (Appendix 1) for quality and cultural adaptability to the Saudi dialect to be compatibly used in the Saudi culture. This evaluation was conducted by two dentistry experts who were bilingual in Arabic and English, with Arabic as their native language. There was no disagreement between the translators. An inclusive pilot study on 30 participants was conducted to assess the clarity of the questionnaire. From a total score of 24, participants with >3 scores were classified as having TMD. The English version of the questionnaire is presented in Appendix 1.

Statistical analysis

Data obtained from the questionnaire were analyzed using SPSS version 20.0 for Windows (IBM corp., Armonk, NY, USA). Descriptive statistics were computed for the demographic details of the participants. The study also added the scoring of frequencies for the 17 questions of the questionnaire. Spearman's correlation coefficients were used to examine the correlation between the symptoms of sleep bruxism, jaw pain, and the limitation in jaw movement. Cronbach’s alpha and interclass correlation coefficients were checked for internal consistency and reliability of the items, respectively. Cronbach's alpha coefficient values above 0.8 indicated good or excellent internal consistency for the questions. 

Validity and reliability

To check the internal consistency of the 17 items of the questionnaire, Cronbach's alpha was carried out; the value of each item was above 0.8, which showed that questionnaire consistency was excellent and can be used to carry out the study. For the validity of the questionnaire convergent validity analysis was done by analyzing TMD and sleep bruxism. For face validation of the questionnaire, the study evaluated the Arabic translation of the questionnaire (Appendix 1) for quality and cultural adaptability to the Saudi dialect to be compatibly used in the Saudi culture. This evaluation was conducted by two dentistry experts who were bilingual in Arabic and English, with Arabic as their native language. There was no disagreement between the translators. An inclusive pilot study on 30 participants was conducted to assess the clarity of the questionnaire. From a total score of 24, participants with >3 scores were classified as having TMD. The English version of the questionnaire is presented in Appendix 1.

## Results

We obtained a total of 200 completed questionnaires. Of these, the study included 170, making the response rate 85%. Table 1 shows the demographics of the participants. The majority of participants were female (54.7%). Around 68.8% (117) were in the age group of 10-14 years. Almost 95.9% of participants' parents were employed, and 70 (41.2%) had the educational status of college level.

**Table 1:** Demographics of participants

Variables		N (%)
Age	10-14	117 (68.8)
	15-19	53 (31.2)
Gender	Male	77 (45.3)
	Female	93 (54.7)
Parent’s employment status	Yes	163 (95.9)
	No	7 (4.1)
Parent’s education status	Below college	32 (18.8)
	College	70 (41.2)
	Postgraduate	68 (40)

**Figure 1:**
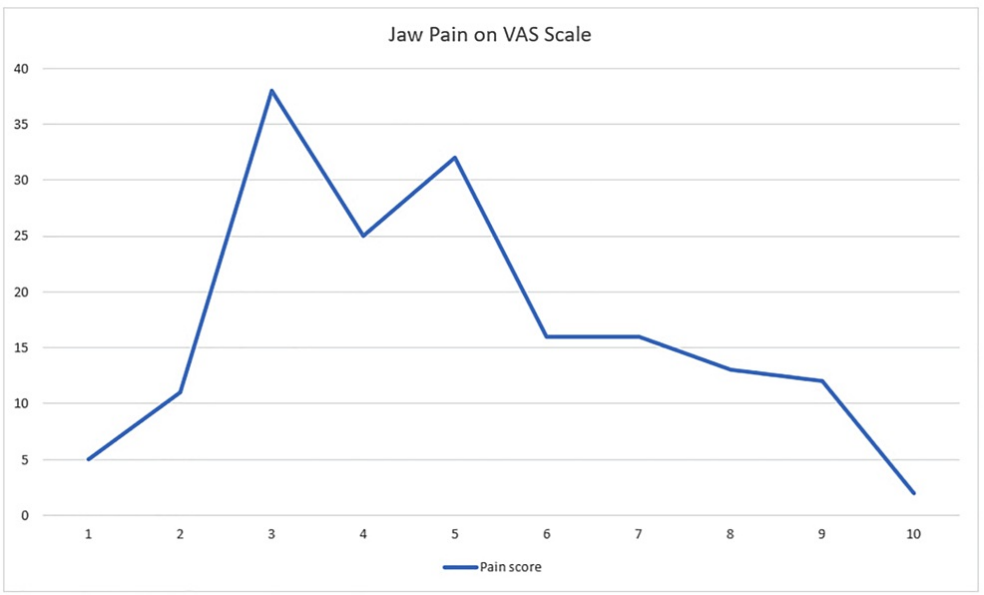
Jaw pain on VAS VAS, visual analog scale

Table 2 represents the responses for sleep bruxism. Around 94 (55.3%) experienced teeth grinding at night, and 95 (55.9%) had dentition worn down more than usual. Those with these two symptoms and at least one sign upon awakening were considered for sleep bruxism, i.e., 90 (52.9%). Regarding jaw lock, the symptoms ranged between 20% and 59.4%. Figure 1 demonstrates the severity of jaw pain on the VAS.

**Table 2:** Questions regarding sleep bruxism and jaw lock

Regarding sleep bruxism	N (%)
Are you aware, or has anyone heard you grinding your teeth frequently during sleep?	94 (55.3)
Are you aware that your dentition is worn down more than it should be?	95 (55.9)
Symptoms upon awakening	
A sensation of fatigue, tightness, or soreness of the jaw upon awakening	101 (59.4)
Feeling that your teeth are clenched or that your mouth is sore upon awakening	74 (43.5)
Aching of your temples upon awakening	78 (45.9)
Difficulty in opening your mouth wide upon awakening	61 (35.9)
Feeling of tension in your jaw joint upon awakening and feeling as if you have to move your lower jaw to release it	63 (37.1)
Hearing or feeling a ‘‘click’’ in your jaw joint upon awakening that disappears afterward	34 (20)
Sleep bruxism	90 (52.9)

Table 3 represents the responses for TMD. Here, the highest felt symptom was a headache, whereas the least people felt clenching of teeth in worry. A total of 76 (44.7%) patients had a score of ≤3 and hence did not have TMJ; comparatively, 94 (55.3%) had a total score >3 and were diagnosed with TMJ. Table 4 shows a significant association between sleep bruxism and TMD (p-value = 0.25). Fifty-seven (63.3%) patients with sleep bruxism also had TMD.

**Table 3:** Scores for TMD TMD, temporomandibular disorder

Regarding TMD		Never	Sometimes	Often	Always
Do you have a symptom such as a headache?	N	11	57	68	34
	%	6.5	33.5	40	20
Do you have a symptom such as pain during mouth closure and opening?	N	90	48	25	7
	%	52.9	28.2	14.7	4.1
Do you have symptoms of joint trismus when getting up in the morning?	N	98	45	22	5
	%	57.6	26.5	12.9	2.9
Do you have symptoms of pain around the neck?	N	92	43	20	15
	%	54.1	25.3	11.8	8.8
Do you have symptoms of tinnitus?	N	102	48	18	1
	%	60.0	28.2	10.6	0.6
Do you clench your teeth when in worry?	N	107	50	13	0
	%	62.9	29.4	7.6	0.0
Do you clench your teeth when in anger?	N	95	39	33	3
	%	55.9	22.9	19.4	1.8
Do you clench your teeth when concentrating?	N	105	46	5	1
	%	61.8	27.1	7.6	2.9
Total score ≤3: Non-TMD	N	76			
	%	44.7			
Total score >3: TMD	N	94			
	%	55.3			

**Table 4:** Association of sleep bruxism and TMD TMD, temporomandibular disorder

		Sleep bruxism		Chi-square p-value (X^2^, Df)
		Yes	No	0.025 (5.000, 1)
TMD	Yes	57 (63.3)	37 (46.3)	
	No	33 (36.7)	43 (53.8)	

Table 5 shows Spearman's correlation coefficients between bruxism, jaw lock, and jaw pain. There was a weak association between jaw pain and jaw lock (coefficient value = 0.249) and bruxism (coefficient value = 0.287), indicating that increased pain was not dependent on growth in jaw lock or bruxism symptoms. Bruxism and jaw lock had a robust correlation (coefficient value = 0.920), indicating an increase in sleep bruxism, with a high chance of jaw lock. 

**Table 5:** Correlation of sleep bruxism, jaw lock, and jaw pain

		Jaw pain	Bruxism
Jaw lock	Correlation coefficient	0.249	0.920
	Significance (two-tailed)	0.001	0.000
Bruxism	Correlation coefficient	0.287	1
	Significance (two-tailed)	0.000	-

## Discussion

This study aimed to validate the sleep bruxism questionnaire regarding the experience of jaw pain and limitation of jaw movement in Saudi Arabian adolescents. The number of female participants was slightly more than males, and most participants were in the age group of 10-14 years. TMD and sleep bruxism were significantly associated. We found a weak correlation between bruxism and jaw pain and a strong correlation with jaw lock. The most experienced symptom was a sensation of fatigue, tightness, or soreness of the jaw upon awakening. Upon awakening, the least experienced symptom was hearing or feeling a ''click'' in the jaw joint that disappears afterward. Similar to the findings, van Selms et al. [19] found symptoms of pain and tightness on awakening in sleep bruxism (OR = 1.47; 95% CI = 1.17-1.86).

Similarly, the “clicking” sound was present in fewer individuals (OR = 1.31; 95% CI = 1.03-1.65). The most experienced TMD symptom was a headache, and the least experienced was a clench of teeth when in worry. According to Silva et al. [20], more people coming to the dental clinic had headaches than those going to the medical centers, implying that TMDs are significant comorbidity of migraine headaches; therefore, it becomes difficult sometimes to differentiate them from tension headaches. This finding may be the reason for the high number of participants reporting headaches. Similarly, Branco et al. [21] also found an association between TMJ disorders and headaches in their study of adolescents and children aged 6-14 years. 

However, this association was not dependent on age or gender [21]. Participants reported clenching teeth more in anger than when concentrating or in worry. Oliveira et al. [22] found a strong association between anxiety and sleep bruxism in children. They found more anxiety traits in children with sleep bruxism than those without it. This study found bruxism incidents in participants with the anger trait than with the worry trait [22]. A recent study [9] showed that jaw lock and sleep bruxism are highly correlated. In this study, 52.9% had sleep bruxism, similar to a previous study in the English version, which found 54% self-reported sleep bruxism in adults [22].

Furthermore, sleep bruxism was not associated with the severity of jaw pain. These results are also similar to a recent study by Smardz et al. [23], in which the intensity of TDM-related pain did not correlate with the power of sleep bruxism. Therefore, pain associated with TDM may help determine TDM disorders. This study does not support pain severity as an indicator of sleep bruxism.

For this questionnaire to be validated, it required reliability and validity. Reliability is the standard by which an assessment tool gives consistent and stable results [24], whereas the validity of a questionnaire is determined when it assesses the desired outcome efficiently [25]. This questionnaire had good-to-excellent reliability based on Cronbach's alpha coefficient. At the same time, convergent validity analysis was done by analyzing TMD and sleep bruxism. Other instruments, such as electromyography, clinical examination, or gold standard test polysomnography (PSG), were not used for assessment as they are expensive and time-consuming and pose challenges in the public health system. In PSG, the placement of electrodes on the face and body of the subject during sleep can be affected by environmental bias [26].

Tooth-grinding sounds do not always occur during rhythmic masticatory muscle activity episodes. However, questionnaires have the drawback that they depend on the self-awareness of the respondents for teeth grinding [27]. The main study limitation was that it included dental patients only and had a convenient sample. Therefore, caution should be applied when generalizing these findings to a different population. Random sampling was challenging due to the unavailability of a complete database of patients visiting the clinics. For generalizability, further study on other clinical and age groups is required.

Similarly, the same questionnaire should be used on adolescent patients in other Saudi Arabian clinics for better external validity. Furthermore, the reported data were collected by self-reporting; therefore, the questionnaire-specific method variance may have been inflated by the relationship between the variables. Also, follow-up of the patients was not possible because it was a cross-sectional study. Further research should include an undertaking by patients for a follow-up to assess other outcomes. 

## Conclusions

 It is essential to standardize this Arabic version to be better understood by different cultures and nations that speak the Arabic language. The Arabic version of the sleep bruxism questionnaire is reliable and valid for assessing sleep bruxism based on the experience of jaw pain and limitation of jaw movement in Saudi Arabian adolescent patients. By validating this questionnaire in other Arabic cultural settings and countries, adapting this questionnaire would benefit cross-cultural research. In conclusion, this proposed Arabic version of sleep bruxism based on the experience of jaw pain and jaw movement limitation in Saudi Arabian adolescents has adequate internal consistency and criterion and convergent validity.

## Figures and Tables

**Figure 2:**
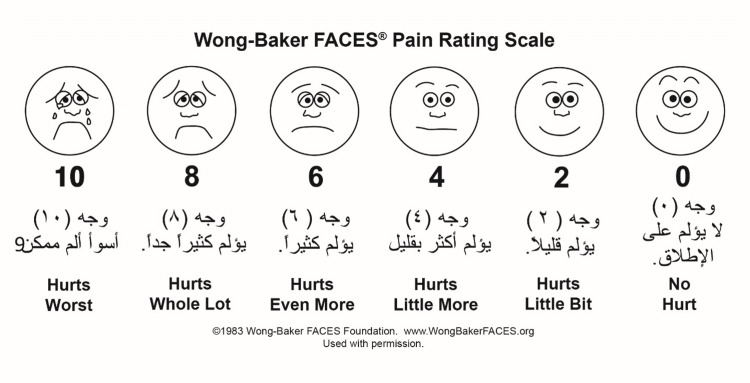
Wong-Baker FACES

**Figure 3:**
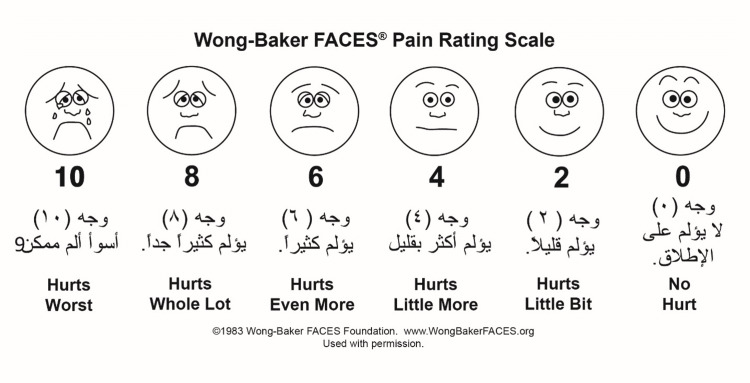
Wong-Baker FACES

## Appendices

Arabic version:

استبيان 

الجزء 1: الديموغرافيات

1. العمر: __________________

2. الجنس:

أ. الذكر

ب. أنثى

الجزء 2: فيما يتعلق بصريف الأسنان (صرير تطاحن الاسنان)

1. هل تعلم ، أو هل سمعك أحد ، تطحن أسنانك كثيرًا أثناء النوم؟

أ. نعم

ب. لا

2. هل تدرك أن أسنانك تآكلت أكثر مما ينبغي؟

أ. نعم

ب. لا

هل أنت على علم بأي من الأعراض عند الاستيقاظ من السؤال 3 إلى 8؟

3. الإحساس بالتعب أو الضيق أو الألم في فكك عند الاستيقاظ؟

أ. نعم

ب. لا

4. تشعر بأن أسنانك مطبقة أو أن فمك يؤلمك عند الاستيقاظ؟

أ. نعم

ب. لا

5. ألم معابدك عند الاستيقاظ؟

أ. نعم

ب. لا

6. صعوبة في فتح فمك واسعا عند الاستيقاظ؟

أ. نعم

ب. لا

7. الشعور بالتوتر في مفصل الفك عند الاستيقاظ والشعور كما لو كان عليك تحريك فكك السفلي لتحريره؟

أ. نعم

ب. لا

8. تسمع أو تشعر "بنقرة(فرقعة)" في مفصل الفك عند الاستيقاظ وتختفي بعد ذلك؟

أ. نعم

ب. لا

9. على WBFPS ما هي درجة ألم فكك.

الجزء 3: بخصوص انظمة الدفاع الصاروخى التكتيكى (خلل المفصل الصدغي الفكي)

1. هل لديك أعراض مثل الصداع؟

أ. أبدا

ب. بعض الأحيان

ج. غالبا

د. دائما

2. هل لديك أعراض مثل الألم أثناء غلق وفتح الفم؟

أ. أبدا

ب. بعض الأحيان

ج. غالبا

د. دائما

3. هل تعاني من أعراض ارتعاش المفاصل (كزاز مفصل الفك) عند الاستيقاظ في الصباح؟

أ. أبدا

ب. بعض الأحيان

ج. غالبا

د. دائما

4. هل لديك أعراض آلام حول الرقبة؟

أ. أبدا

ب. بعض الأحيان

ج. غالبا

د. دائما

5. هل لديك أعراض طنين الأذن؟

أ. أبدا

ب. بعض الأحيان

ج. غالبا

د. دائما

6. هل تضغط على أسنانك عندما تكون في قلق؟

أ. أبدا

ب. بعض الأحيان

ج. غالبا

د. دائما

7. هل تقبض (تضغط على) أسنانك عند الغضب؟

أ. أبدا

ب. بعض الأحيان

ج. غالبا

د. دائما

8. هل تضغط على أسنانك عند التركيز؟

أ. أبدا

ب. بعض الأحيان

ج. غالبا

د. دائما

رموز الأرقام للجزء 3

(أبدًا = 0 ، أحيانًا = 1 ، غالبًا = 2 ، دائمًا = 3)

مجموع النقاط: 0-24

مجموع النقاط ≤ 3: اضطراب غير الفك الصدغي

مجموع النقاط> 3: اضطراب الفك الصدغي

English version:

Part 1: Demographics

1.     Age: __________________                             

2.     Gender:

a.     Male

b.     Female

Part 2: Regarding Sleep Bruxism

1.     Are you aware, or has anyone heard you, grinding your teeth frequently during sleep?

a.     Yes

b.     No

2.     Are you aware that your dentition is worn down more than it should be?

a.     Yes

b.     No

Are you aware of any of the symptoms upon awakening from Question 3 to 8?

3.     Sensation of fatigue, tightness or soreness of your jaw upon awakening?

a.     Yes

b.     No

4.     Feeling that your teeth are clenched or that your mouth is sore upon awakening?

a.     Yes

b.     No

5.     Aching of your temples upon awakening?

a.     Yes

b.     No

6.     Difficulty in opening your mouth wide upon awakening?

a.     Yes

b.     No

7.     Feeling of tension in your jaw joint upon awakening and feeling as if you have to move your lower jaw to release it?

a.     Yes

b.     No

8.     Hearing or feeling a ‘‘click’’ in your jaw joint upon awakening that disappears afterwards?

a.     Yes

b.     No

9.     On WBFPS what is the score of your jaw pain?

Part 3: Regarding TMD

1.     Do you have symptom such as headache?

a.         Never

b.         Sometimes

c.         Often

d.         Always

2.     Do you have symptom such as pain during closing and opening mouth? 

a.         Never

b.         Sometimes

c.         Often

d.         Always

3.     Do you have symptom of joint trismus when getting up in the morning? 

a.         Never

b.         Sometimes

c.         Often

d.         Always

4.     Do you have symptom of pain around neck? 

a.         Never

b.         Sometimes

c.         Often

d.         Always

5.     Do you have symptom of tinnitus? 

a.         Never

b.         Sometimes

c.         Often

d.         Always

6.     Do you clench your teeth when in worries? 

a.         Never

b.         Sometimes

c.         Often

d.         Always

7.     Do you clench your teeth when in anger? 

a.         Never

b.         Sometimes

c.         Often

d.         Always

8.     Do you clench your teeth when concentrating?

a.         Never

b.         Sometimes

c.         Often

d.         Always

Number codes for Part 3 (never = 0, sometimes = 1, often = 2, always = 3)

Total score: 0-24 

Total score ≤3: Non-temporomandibular disorder 

Total score >3: Temporomandibular disorder
